# Blindfolded hypogravity adaptation differentially affects motor and cognitive systems

**DOI:** 10.3389/fpsyg.2026.1729003

**Published:** 2026-02-18

**Authors:** Chase G. Rock, Hyorim Kim, Joelle F. Dick, Young-Hui Chang

**Affiliations:** Comparative Neuromechanics Laboratory, School of Biological Sciences, Georgia Institute of Technology, Atlanta, GA, United States

**Keywords:** biomechanics, gravity adaptation, locomotion, motor control, motor learning, motor-cognitive, vision

## Abstract

**Introduction:**

Motor adaptation is essential for human movement and is strongly influenced by visual feedback as evidenced by motor and cognitive aftereffects following visuomotor adaptation. In some cases, these aftereffects are also transferred to related motor or cognitive tasks. When vision is lost or disrupted, motor adaptation must be accomplished by other sensory modalities, such as proprioception, but the degree of transfer following such adaptation is unclear. The aim of the current study was to determine the necessity of vision for motor adaptation and subsequent transfer to a cognitive task.

**Methods:**

We leveraged a previously developed paradigm for studying motor and cognitive aftereffects due to adaptation to jumping in simulated hypogravity. We tested 15 participants’ jump performance in 1 g, along with their performance on a gravity-based cognitive task, before and after they performed targeted jumps in simulated hypogravity. Crucially, participants were blindfolded during all jumps, relying on provided audio cues and proprioception instead of visual feedback.

**Results:**

Despite the lack of vision, we observed the hallmarks of hypogravity motor adaptation—muscle preactivation was reduced and jump height was reduced after returning to normal gravity. However, no aftereffects were observed in the cognitive task.

**Discussion:**

Therefore, it appears that vision is not necessary for successful adaptation to simulated hypogravity, but vision may be necessary for the subsequent transfer of aftereffects to the cognitive task.

## Introduction

Motor adaptation is a critical feature of human movement, allowing for a diverse motor repertoire in the face of an ever-changing environment. Even when people are faced with unnatural phenomena—displaced vision through prism goggles (Harris, 1963), novel forcefields (Lackner and Dizio, 1994; Shadmehr and Mussa-Ivaldi, 1994), treadmills that move either foot at different speeds (Malone et al., 2012; Finley et al., 2014; Selgrade et al., 2017)—they show remarkable adaptability. This adaptability is facilitated by sensory feedback, which can directly impact muscle action (Nichols and Houk, 1973) as well as inform cognitive strategies and perceptions related to movement (Torok et al., 2019; Bear et al., 2022). In considering which specific sensory modalities are important for the motor and cognitive aspects of motor adaptation, vision is undoubtedly a key contributor.

It is difficult to overstate the importance of vision in motor adaptation. Many everyday tasks are visually guided, and many motor adaptation paradigms are built around the manipulation of visual feedback: for example, prism-shifted vision (Harris, 1963; Morton and Bastian, 2004; Bakkum et al., 2020), rotated visual feedback (Taylor et al., 2014; Miller-Mills et al., 2024; Tsay et al., 2024), changes in optic flow (Warren et al., 2001; Solini et al., 2021; Lin and Linkenauger, 2024). Vision is especially important for informing cognitive predictions and expectations related to movement. For example, a person’s expectation of required lift force is strongly influenced by the visually perceived size of the object, known as the size-weight illusion (Charpentier, 1891; Murray et al., 1999). This illusion can even persist uncorrected when vision is occluded during the actual lifts, despite tactile experience with the object (Buckingham and Goodale, 2010), providing a strong example of the power of vision over feedforward, movement-related expectations.

The necessity of vision for motor adaptation in specific tasks can vary. Other sensory modalities, like proprioception (sensation pertaining to body-sense (Sherrington, 1906)), can facilitate motor adaptation, especially when vision is unavailable or unreliable (DiZio and Lackner, 2000; Franklin et al., 2007). In fact, feedforward motor strategies can even be maintained when vision is entirely absent, such as when people jump from an unknown height without visual feedback (Magalhães and Goroso, 2009, 2011), indicating that vision is not required for certain types of adaptation and other sensory modalities can accommodate its absence. However, when all sensory modalities can contribute, it is difficult to distinguish which sensory modality is driving motor adaptation. For example, recent work from our group has shown updated gravity-related expectations following an adaptation to hypogravity jumping (Rock et al., 2024), but we were unable to determine the sensory influences on this adaptation.

Specifically, upon returning participants to normal gravity, aftereffects were observed in jumping, arm-movements, and cognitive perceptions related to gravity (Rock and Chang, 2024; Rock et al., 2025). Action aftereffects in targeted jumping at 1 g following hypogravity exposure appeared as reduced muscle preactivation prior to landing and increased vertical target error. Subsequent experiments found aftereffects in other gravity-related tasks following hypogravity jumping, including an upper body action task (vertical reaching) and a non-action task (cognitive assessment of projectile motion). However, because vision and all other sensory modalities were unrestricted during these experiments, we could not say for certain which sensory modalities are sufficient and/or necessary for the aftereffects in action and non-action tasks to occur. Therefore, a modified version of this hypogravity jumping paradigm that selectively disrupts vision may provide a unique opportunity to determine how vision contributes to the motor and cognitive aftereffects that result from motor adaptation. As stated above, vision is important for many types of motor adaptation, but is not strictly required for others. Therefore, our approach will allow us to answer two questions: (1) Is vision required during hypogravity adaptation for aftereffects to appear in an action task? and (2) Is vision required during hypogravity adaptation for aftereffects to appear in a non-action task?

## Materials and methods

### Participants

Fifteen participants (8 female, 7 male; height: 171.0 ± 9.7 cm; mass: 64.8 ± 16.7 kg; age: 22.0 ± 3.8 years) provided informed consent according to a protocol approved by the Georgia Institute of Technology Institutional Review Board (Protocol #H19325). Fourteen participants self-reported that they were right-handed and one reported left-hand dominance. By observing the foot each participant used to kick a ball (van Melick et al., 2017), we determined that 14 participants were right-foot dominant and one was left-foot dominant.

### Equipment

Kinematic and kinetic data were collected using a 12-camera 3D motion analysis system (Vicon Motion Systems, Oxford, UK) and two floor-embedded force plates (AMTI, Watertown, MA). An EMG electrode system (Motion Lab Systems, Baton Rouge, LA) was used to measure muscle activity from the dominant and non-dominant side triceps surae group: specifically, the Soleus muscle (SOL), Medial Gastrocnemius muscle (MG), and Lateral Gastrocnemius muscle (LG). Data were collected and synchronized through Vicon Nexus, processed in Visual3D (C-Motion, Germantown, MD), and final calculations and comparisons were performed using custom MATLAB scripts. Hypogravity was simulated through the use of a custom reduced gravity simulator (RGS) that has been described previously (Rock et al., 2024). Participants wore their own shoes throughout the protocol.

### Experimental set-up

The overall procedure for this experiment largely replicated our previous work (Rock et al., 2024). Briefly, the skin overlying each muscle belly was prepped for EMG electrode placement by shaving any hair and rubbing briskly with an alcohol wipe to reduce impedance. EMG electrodes were placed on the SOL, MG, and LG according to previously published guidelines (Konrad, 2005) and tested for signal quality. A retroreflective marker for the live feedback system was placed on the sternal notch, with additional markers placed on vertebra C7, clavicle (left and right side), scapula (left and right side), and/or the neck to facilitate motion tracking of the sternal notch.

Participants first performed three maximum jumps with their arms crossed to minimize any arm movements relative to the body center of mass. Participants were allowed at least 30 s of rest between maximum jumps to minimize the effects of fatigue and allow for restoration of chemical energy stores (Baker et al., 2010). The target height for all subsequent jumps was set at 75% of the maximum vertical displacement during the participant’s highest maximum jump.

All jumps during the experimental protocol were performed blindfolded with their eyes closed and with live audio feedback that consisted of three distinct sounds linked to the vertical position of the sternal marker using Vicon. One sound was a continuously played tone that lowered in pitch with downward movement and increased in pitch with upward movement. The change in pitch was determined by a vertical range of 1.3 m, which was maintained for each participant and used the default sound settings from Vicon Nexus. The second sound played when the participant’s vertical position rose into the target area (2 cm below the target height), triggering a pleasant bell chime that indicated successful achievement of the target height. The third sound played with the participant’s vertical position exceeded the target height by 2 cm, triggering an unpleasant buzzer sound that indicated they had jumped too high. Prior to starting the experimental protocol, all participants were familiarized with these sounds and attested that they could distinguish changes in the continuous tone prior to the jumping protocol.

### Experimental protocol

After all experimental set-up procedures were completed, participants commenced with the experimental protocol. Participants alternated between two tasks during the experimental protocol: a cognitive assessment task and a jumping task.

The cognitive assessment task (Rock and Chang, 2024; Rock et al., 2025a) requires the participants to sit on a stool and view a ball being dropped in a virtual space, displayed on a screen (105 × 60 cm) 3 m in front of them at eye level. The virtual space was a custom-made basketball court that was scaled to match real-life dimensions to provide spatial context to the scene (Unity, Unity Technologies, San Francisco, CA, USA), including the basketball court, basketball hoop (3.05 m above ground), and basketball (24 cm in diameter; Supplementary Figure 1). Using a wireless keyboard, each drop was initiated by the participant, and fell from the same height (4 m above ground), but a random gravity level (ranging from 0.5 to 1.5 g in steps of 0.1 g, including 1 g, resulting in 11 gravity levels) was applied to the ball for each drop. After each drop, the participants indicated whether the ball fell “fast” or “slow” (compared to the participant’s subjective “normal”) using buttons on a computer keyboard (i.e., two-alternative forced choice). The instructions given to the participant stressed that they were to compare to whatever they considered to be “normal”, not to the previous trial or to the average gravity that they observed over the course of several trials. They were also told that the different accelerations would be presented in a random order and that there may or may not be an equal number of fast and slow drops. The cognitive task consisted of 99 trials (9 repetitions of 11 gravity levels) and was performed four times: before the first bout of jumping (Familiarization), before jumping in simulated hypogravity (PRE), immediately after jumping in simulated hypogravity (POST), and once after all jumping had been completed (Washout).

For the jumping task, all jumps were performed with the participant blindfolded. The participants first jumped 10 times at unaltered gravity (Pre Jumps 1–10), with their arms held across their chest toward the goal of reaching the virtual target as accurately as possible. The subjects jumped once for every time a lab member said “Go” and were allowed to rest for as long as needed between jumps. Participants were asked to try to refrain from taking any extra steps before or after the jump. Though not specifically instructed, all participants used a countermovement leading into each jump. During these first 10 jumps, the research team ensured that the participants understood the audio feedback.

After the first set of 10 jumps, the blindfold was removed and the participant performed the cognitive assessment task again (PRE). They then performed 50 blindfolded jumps while attached to the RGS, where hypogravity was simulated by offloading the participant by ~50% of their bodyweight. This was accomplished by a set of constant-force springs mounted above the participant and attached to a body-worn harness (Rock et al., 2024). Immediately upon completing the hypogravity jumps, they were disconnected from the RGS and again performed the cognitive assessment task (POST). They then performed 10 final, blindfolded jumps (Post Jumps 1–10) at normal gravity, followed by a final bout of the cognitive assessment task (Washout).

### Data analysis

A previously reported method (Rock et al., 2024) was used to find the EMG preactivity of the triceps surae muscles for the right and left legs of each participant. Briefly, the time of muscle activity onset prior to landing was determined for each muscle using the filtered (lowpass cutoff at 450 Hz and highpass cutoff at 5 Hz), rectified, and normalized EMG signal by determining the timepoint where the muscles activity rose most rapidly (Santello and McDonagh, 1998). Landing was defined as the first frame where the ground reaction force beneath the participant exceeded 25 N (1,000 Hz; AMTI, Watertown, MA, USA). The muscle activation signal was then integrated between the time of muscle activity onset and landing. The integrated activity from the SOL, MG, and LG muscles were then averaged to provide a summary metric for that leg’s EMG activity prior to landing (i.e., Triceps Surae Preactivity). Jump height was calculated by the vertical displacement of the retroreflective marker attached to the sternum. Target error was calculated by subtracting the target height from the jump height and taking the absolute value.

For the cognitive assessment task, we performed the same analysis procedure as reported previously (Rock and Chang, 2024; Rock et al., 2025). For each participant, the proportion of responses indicating “Fast” was plotted at each tested gravity level and fit with a log–log curve (MATLAB, Natick, MA, USA). A log–log link function was fit to the responses, similar to previous work in estimating falling objects (Moscatelli and Lacquaniti, 2011; Torok et al., 2019), and was then used to find the point of subjective equality (i.e., the gravity level where participants would be equally likely to respond “Fast” or “Slow”). The point of subjective equality was interpreted as the gravity level that the participant was ostensibly comparing to during each bout (Familiarization, PRE, POST, and Washout). The log–log curve was further analyzed by determining the points at which it crossed 0.3 and 0.7. The range in gravity levels that were required to contain these two points was taken as a measure of the participant’s sensitivity to differing gravity levels, similar to previous studies that use the interquartile range 0.25–0.75 (Ostry et al., 2010; Mirdamadi et al., 2024), but as multiple participants in the current study did not reach 0.75 in POST, we reduced the threshold to 0.7 and adjusted the lower bound to match.

### Statistical analysis

An *a priori* power analysis was run on pilot data where participants performed a non-blindfolded version of the protocol (dz = 1.387, alpha = 0.05, desired power = 0.8) revealing a total sample size of 7 participants would provide a power of 0.861 in a two-tailed, paired t-test comparing PRE and POST. Due to the difference in protocol (i.e., blindfold), to account for potential participant attrition, and due to the low-risk nature of the study, we doubled this number and collected 15 participants.

For Triceps Surae Preactivity, jump height, and target error, a one-way, repeated measures ANOVA (alpha = 0.05) was used to compare the final jump prior to hypogravity exposure (Pre Jump 10), the first jump upon returning to normal gravity (Post Jump 1), and the final jump (Post Jump 10). Partial eta squared ( $\eta_{p}^{2}$ ) was used to estimate effect size (Hentschke and Stüttgen, 2011; Lakens, 2013) and post-hoc, pairwise comparisons were made using Fisher’s least significant difference procedure. Assumptions of normality and sphericity were tested using Shapiro Wilk’s test (Öner and Deveci Kocakoç, 2017) and Mauchly’s test, respectively. If either assumption was shown to be violated, Friedman’s test was used in place of ANOVA with Wilcoxon Signed Rank test used for pairwise comparisons and Kendall’s coefficient of concordance (W) for effect size (Conover and Conover, 1999). If outliers were detected (more than 1.5× interquartile range above or below the upper or lower quartile, respectively), that participant’s data were omitted from analysis of that variable.

The same method was used for the cognitive assessment task, with the point of subjective equality and the gravity sensitivity compared across PRE, POST, and Washout conditions.

## Results

**Figure 1:**
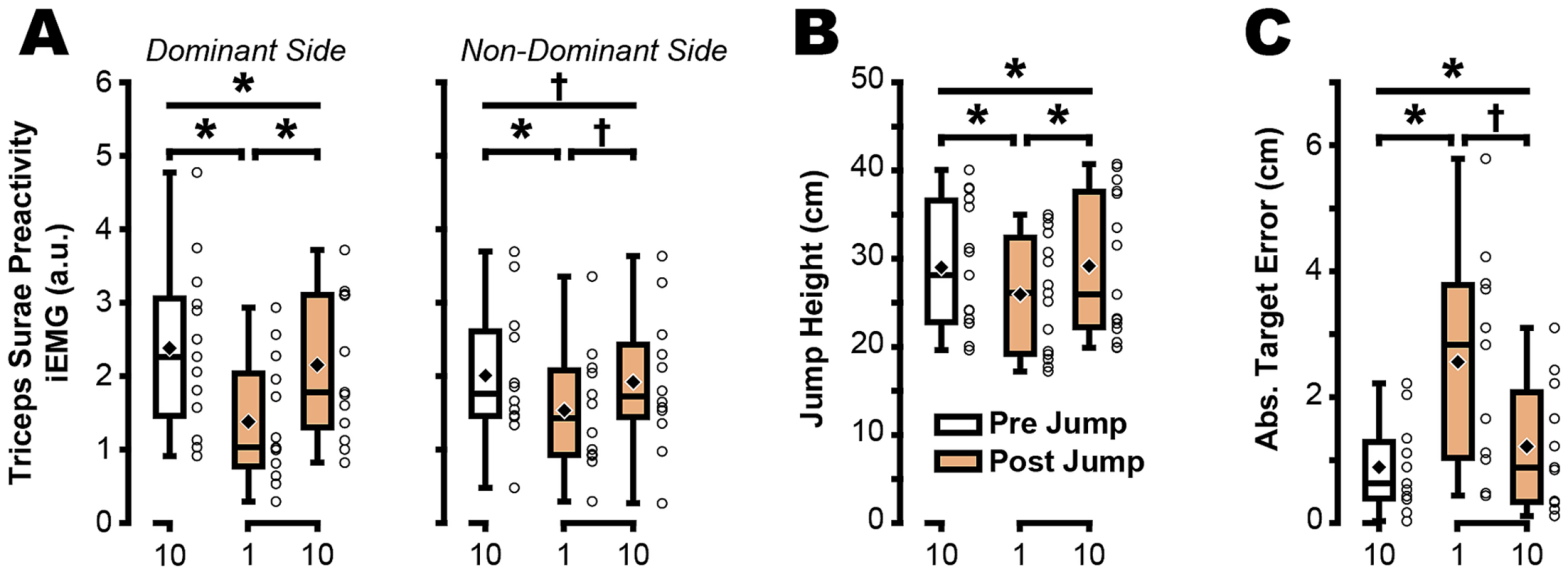
Triceps Surae Preactivity and jumping performance were altered following hypogravity adaptation. Single trial data from the final jump prior to simulated hypogravity exposure (white box) were compared to the first jump and the tenth jump after being removed from the reduced gravity simulator (orange boxes). Triceps Surae Preactivity **(A)**, jump height **(B)**, and absolute target error **(C)** were all affected in the first jump after returning to normal gravity. Boxplots represent the median (horizontal line), mean (black diamond), interquartile ranges (top and bottom of each box), and range (whiskers) across all participants. Open circles represent individual participant data for each trial. * Indicates *p* < 0.05, † indicates *p* < 0.10.

Jump performance upon returning to 1 g was significantly impacted by the exposure to jumping in a lower simulated gravity level (0.48 ± 0.07 g). A main effect of condition was found for Triceps Surae Preactivity in the dominant leg (*F*(2,12) = 24.095, *p* < 0.001, *η^2^_p_ = 0.6675*; Figure 1; Table 1), with post-hoc comparisons showing reduced preactivation in Post Jump 1 when compared with Pre Jump 1 (*p* < 0.001) and when compared with Post Jump 10 (*p* < 0.001). While a main effect of condition was not found for Triceps Surae Preactivity in the non-dominant leg (*F*(2,11) = 3.047, *p* = 0.068, $\eta_{p}^{2}$ = 0.2169; Figure 1; Table 1), the analysis was close to the critical *F*-value of 3.98, so we commenced with post-hoc pairwise comparisons, revealing a potentially reduced preactivation in Post Jump 1 when compared to Pre Jump 10 (*p* = 0.015) and a near-significant difference when compared to Post Jump 10 (*p* = 0.076).

**Table 1:** Statistical summary.

Variable of Interest	df	Test statistic (F-stat or X^2^)	*p*	Effect size ( $\eta_{p}^{2}$ or W)
Triceps Surae Preactivity—dominant side	2,12	24.10	**<0.001**	0.6675
Pre Jump 10 vs. Post Jump 1			**<0.001**	
Pre Jump 10 vs. Post Jump 10			0.152	
Post Jump 1 vs. Post Jump 10			**<0.001**	
Triceps Surae Preactivity—Non-dominant side	2,11	3.047	*0.068*	0.2169
Pre Jump 10 vs. Post Jump 1			**0.015**	
Pre Jump 10 vs. Post Jump 10			0.722	
Post Jump 1 vs. Post Jump 10			*0.076*	
Jump height^np^	2,14	17.73	**<0.001**	0.5910
Pre Jump 10 vs. Post Jump 1			**<0.001**	
Pre Jump 10 vs. Post Jump 10			0.489	
Post Jump 1 vs. Post Jump 10			**0.005**	
|Target error|	2,10	5.772	**0.011**	0.3660
Pre Jump 10 vs. Post Jump 1			**0.015**	
Pre Jump 10 vs. Post Jump 10			0.355	
Post Jump 1 vs. Post Jump 10			*0.054*	
Point of subjective equality	2,13	1.412	0.262	0.0980
PRE vs. POST			0.355	
PRE vs. Washout			0.619	
POST vs. Washout			0.069	
Width of 30%–70% region	2,12	0.175	0.841	0.0144
PRE vs. POST			0.714	
PRE vs. Washout			0.589	
POST vs. Washout			0.859	

np indicates non-parametric tests were used; italics indicate *p* < 0.10 and bold indicates *p* < 0.05.

For jump height, the assumptions required by ANOVA were not met (Mauchly’s test of sphericity *X^2^*(2) = 7.37, *p* = 0.025) and so a Friedman’s test was applied, revealing a main effect of condition (*X^2^*(2) = 17.73, *p* < 0.001, W = 0.5910; Figure 1; Table 1). Pairwise comparisons were made using a Wilcoxon Signed Rank test and revealed a significantly lower jump height in Post Jump 1 when compared to Pre Jump 10 (*p* < 0.001) and when compared to Post Jump 10 (*p* = 0.005). For target error, a main effect of condition was found (*F*(2,10) = 5.772, *p* = 0.011, *η^2^_p_ = 0.3660*; Figure 1; Table 1). Pairwise comparisons revealed an increased target error in Post Jump 1 when compared to Pre Jump 10 (*p* = 0.015), and a near-significant increase in target error in Post Jump 1 when compared to Post Jump 10 (*p* = 0.054).

**Figure 2:**
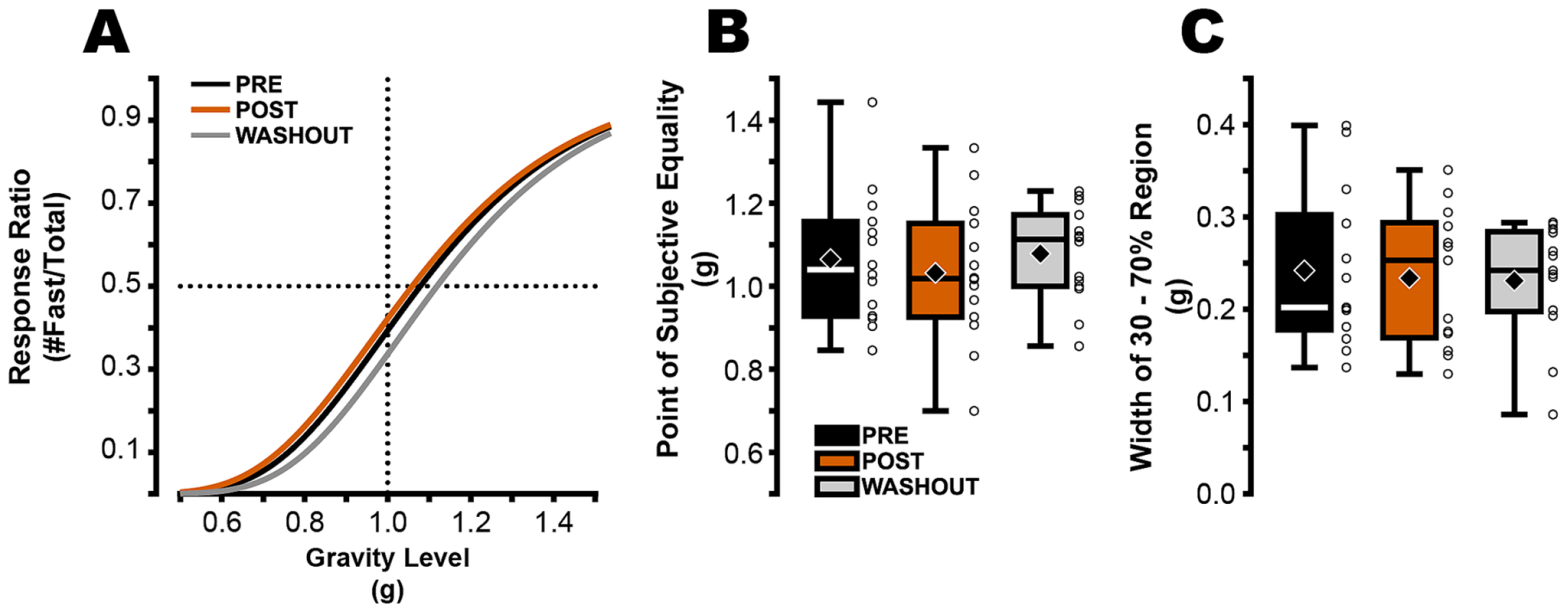
The perception of “normal” gravity acting on the virtual ball was not altered following jumping in simulated hypogravity. **(A)** Psychometric curves (average across subjects) were constructed based on participant responses in PRE (black), POST (orange), and washout (gray) to determine the point of subjective equality (i.e., the gravity level at which participants were equally likely to respond “Fast” or “Slow”). The horizontal dotted line intersects each curve at its point of subjective equality and the vertical dotted line highlights *g* = 1 (or −9.81 m/s^2^). **(B)** Upon returning to normal gravity, no significant difference in PSE was observed between PRE and POST or between PRE and washout. **(C)** The width of the gravity range of the psychometric curve that is bounded by 0.3 and 0.7 response ratios provides a measure of the slope of the psychometric curve. No statistically significant differences were observed between PRE and POST or between PRE and washout (Boxplot description provided in Figure 1).

For the cognitive task, there was no main effect of condition on the point of subjective equality (*F*(2,13) = 1.412, *p* = 0.262, $\eta_{p}^{2}$ = 0.0980; Figure 2; Table 1). In PRE, the point of subjective equality was 1.07 ± 0.15 g (Figure 2). In POST, the point of subjective equality was 1.05 ± 0.17 g (*p* = 0.571; Figure 2; Table 1). Similarly, participants’ ability to discriminate fast and slow trials was not impacted by blindfolded hypogravity exposure, with no main effect of condition on the width of the log–log curved bounded by 0.30 and 0.70 (*F*(2,12) = 0.175, *p* = 0.841, $\eta_{p}^{2}$ *=* 0.0144; Figure 2; Table 1).

## Discussion

### Vision is not required for motor adaptation to hypogravity

Humans are skilled at interacting with the effects of gravity, which is exemplified by quick and effective adaptation to different gravity levels and even different body orientations relative to gravity (Cavagna et al., 1998; Crevecoeur et al., 2009; Gaveau et al., 2014; White et al., 2020). Much of the work in gravity adaptation attributes this adaptation to the central nervous system, wherein an internal model of gravity is trained and accessed for gravity-related action and non-action tasks (Bosco et al., 2008; Zago et al., 2008; Rousseau et al., 2021). The aim of the current study was to determine the involvement of vision in gravity adaptation of an action task and subsequent transfer to a non-action task. In line with our first hypothesis, the results of the current study show that motor adaptation to jumping in hypogravity occurs even in the absence of vision. More specifically, the reduction in Triceps Surae Preactivity after returning to normal gravity revealed a continued expectation of hypogravity, similar to when the same protocol was performed with full vision (Rock et al., 2024). This reduction in preactivation was accompanied by reduced jump height and increased target error, which are also indicative of an expectation of hypogravity as less vertical force was put into the jump. From a methodology perspective, the consistency of the current study’s motor aftereffects with previous iterations of the protocol indicates that the substitution of experimentally provided visual feedback with auditory feedback was largely successful. However, it should be noted that the aftereffect was not entirely symmetrical, with no significant main effect in the non-dominant leg, which differs from our previous findings that show bilateral jumping aftereffects (Rock et al., 2024)^1^. Though the effect in the non-dominant leg was close to significance (*p* = 0.068), a difference in the aftereffect across the legs in this iteration of the paradigm would indicate that vision differentially affects the control of the dominant and non-dominant legs, with more impact on the control of the dominant limb. Such a finding might reflect previous work in handedness, where removal of visual feedback appears to affect the dominant hand more than the non-dominant hand (Przybyla et al., 2013). Despite the asymmetry in the current study, the observed aftereffects in the action task of jumping indicate that vision may not be required to drive hypogravity motor adaptation when feedback from other sensory systems is available.

Previous investigations into jumping adaptation have recognized that, while vision is an important sensory input for adaptation, adaptation can still occur when vision is occluded. Much of the research on muscle preactivation has employed drop jumps and drop landings, where participants jump down from specific heights (Bubeck and Gollhofer, 2001; Lazaridis et al., 2010; Ambegaonkar et al., 2011). For example, when participants were blindfolded and jumped from an unknown height, the timing of their muscle preactivity was abnormal. However, with repeated jumps from the same height—all performed while blindfolded—the muscle preactivity timing quickly matched non-blindfolded values (Magalhães and Goroso, 2009). Notably, when the same protocol was performed by people with long-term blindness, they did not show the initial muscle preactivation “error” even in the first drop; in fact, their preactivation values matched those of sighted people with full vision (Magalhães and Goroso, 2011). The current study extends these findings by showing that adaptation to a new gravity level, as opposed to an enforced changed in drop height, can occur in the absence of vision, as shown in the substantial aftereffect in preactivation upon returning to normal gravity.

### Vision is a prerequisite for cognitive aftereffects to manifest

In contrast, we reject our second hypothesis as the results of the current study make it clear that, although motor adaptation to hypogravity occurred relatively unhindered in the absence of vision, the subsequent aftereffects in the cognitive-perceptual task did not appear. This is despite the previous observance of aftereffects in this non-action task following jumping in hypogravity with vision fully available (Rock and Chang, 2024; Rock et al., 2025). For example, participants who went through the same protocol as the current study, but without the blindfold (i.e., full vision throughout the protocol), saw a significant cognitive aftereffect in POST) (see text footnote 1, respectively). More specifically, the average PSE in the cognitive task went from 1.04 g to 1.12 g in POST. However, the participants in the current study did not experience this effect. So, why is vision required during motor adaptation for transfer to the cognitive task?

From a physiological perspective, for hypogravity adaptation to transfer from the motor to the cognitive task, the same sensory modality may need to be employed for both tasks. The cognitive task was a purely visual task: there were no auditory, proprioceptive, or other sensory cues. In contrast, by removing visual feedback from the jumping task, its adaptation was accomplished through the remaining sensory modalities of auditory, vestibular, and somatosensory feedback. This is similar to previous observations in drop paradigms where movement was successfully adapted despite altered/absent visual feedback (Vidal et al., 1979; Liebermann and Goodman, 2007; Magalhães and Goroso, 2009). While intersensory transfer has previously been observed within a task [e.g., audio/visual object identification (Yildirim and Jacobs, 2015)], perhaps the action and non-action tasks in the current study were too different for such transfer to occur. Therefore, without vision being specifically entrained in the adaptation task, aftereffects in the visually mediated cognitive task were hindered.

From a psychophysical perspective, the hypogravity jumping adaptation in the current study may be largely egocentric, limiting its ability to transfer to the exteroceptive cognitive task. Sensory modalities can provide information about the state of one’s body, about the environment, or both. Vision, as well as many other senses (e.g., gustation, audition, touch), are primarily exteroceptive—they pertain primarily to sensing one’s environment. In contrast, proprioception largely deals with sensing the position and movement of one’s own body through sensors in the joints, muscles, and tendons (Sherrington, 1906). When vision is fully available for hypogravity adaptation, as it was in previous protocols (Rock et al., 2024), it was able to provide the exteroceptive feedback in conjunction with the proprioceptive adaptation. In adapting to blindfolded hypogravity jumping, adaptation was accomplished with reduced exteroceptive feedback and unhindered proprioceptive feedback, possibly leading to a reweighting of the sensory modalities to prioritize proprioception and deprioritize vision (Ernst and Banks, 2002). Without vision, the connection between exteroceptive, environmental perception and proprioceptive, ego-centric perception was perhaps more tenuous. Therefore, when participants were tested in a purely exteroceptive task (i.e., the cognitive-perceptual task), the aftereffects due to a primarily interoceptive adaptation (i.e., blindfolded hypogravity jumping) were absent.

### Informing a multisensory account of hypogravity adaptation

Vision is relied on for many activities of daily life, facilitating precise movement and appropriate motor planning. Recall the last time you navigated a room in total darkness—your movements were likely slower and more careful than if you had full vision. The role of vision in motor control and adaptation has been of interest for decades, with deficits in vision commonly leading to deficits in motor performance (Harris, 1963; Lackner and Lobovits, 1977; Vidal et al., 1979; Patla, 1997). Therefore, it is reasonable to expect that entirely occluding vision would lead to diminished cognitive-motor adaptability. Though we should note that, in some cases, the removal of vision can actually improve adaptation, such as adaptation to split-belt walking (Torres-Oviedo and Bastian, 2010). Additionally, though commonly thought of as separate, the motor and cognitive systems have significant functional and neural overlap. For example, language comprehension—an ability that may appear to belong fully to the cognitive realm—is facilitated by the motor cortex when interpreting action words (Hauk et al., 2004; Pulvermüller, 2005). When it comes to gravity adaptation, both motor actions (Chang et al., 2000; McIntyre et al., 2001; Gaveau et al., 2014; Lacquaniti et al., 2017; Rock et al., 2024) and cognitive-perceptual behavior (Torok et al., 2019) have separately been observed to adapt to different gravity conditions. Recently, we showed that when vision is fully available, adaptation to hypogravity jumping can transfer to gravity-dependent arm movements as well as to the cognitive-perceptual task employed in the current study (Rock and Chang, 2024; Rock et al., 2025), perhaps via an updated internal model of gravity. In fact, there is a body of evidence supporting an internal model of gravity, localized to the temporo-parietal junction, that impacts both visual perception and motor actions (Zago et al., 2005, 2008; Bosco et al., 2008; Rousseau et al., 2021). However, the role of vision in previous research and in our recent observations had been difficult to assess without specifically targeting vision.

The current study aimed to fill these gaps by determining how the absence of vision affects motor-cognitive adaptation to hypogravity. We found that motor aftereffects emerged even in the absence of vision, but cognitive aftereffects did not, supporting a dissociation between the motor and cognitive systems for their respective action and non-action tasks. However, there are some methodological limitations to consider; for example, though 50 jumps appear to be sufficient for motor adaptation, perhaps more are needed for blindfolded, cognitive adaptation. Additionally, it is possible that there is some small effect that we were unable to detect with the current sample. For example, perhaps a variation in relevant life experience (e.g., ball sports, physics-based computer games) increased the variability in PSE and future work may minimize this variability by recruiting more homogenous samples. Also, the blindfold and audio feedback may affect the participants in unanticipated ways, such as changing their emotional state in ways that potentially impact adaptation. Most importantly, the lack of a full-vision control group in the current study prevents drawing a clear-cut, causal conclusion about the role of vision in gravity adaptation. Future work is needed to confirm these results by more directly comparing adaptation across different sensory conditions. Even with these limitations in mind, these results call into question whether a centralized internal model of gravity is employed for all gravity-dependent tasks, regardless of their end-effector. Instead, it appears that the overlap of gravity-dependent tasks is more complex, informed by recent gravity-related experience but also modulating the impact of that experience based on the sensory modalities employed. This is further complicated by the existence of multiple neural streams for visual information which differently affect action and perception (Binkofski and Buxbaum, 2013). Additionally, aging (from early development to advanced age) and disease states see different rates of change in sensory, motor, and cognitive systems (Simion and Butterworth, 1998; Owsley, 2011; Auclair-Ouellet et al., 2017), presenting at once a goal for improving people’s daily lives and an opportunity for dissociating the effects of different systems. Further investigations would benefit from a comprehensive assessment of the sensory systems affected by and involved in hypogravity adaptation and related gravity-dependent tasks.

## Data Availability

The raw data supporting the conclusions of this article will be made available by the authors, without undue reservation.

## Footnotes

1 Rock, C. G., Yang, X., Luo, A., Kim, H., Singh, T., Dick, J., et al (2025). Transfer of gravity adaptation between action and non-action tasks (Under Review).
