# Supplementary material for: Blindfolded hypogravity adaptation differentially affects motor and cognitive systems
Source: Front Psychol. 2026 Feb 18;17:1729003. doi: 10.3389/fpsyg.2026.1729003 (PMC12956515; doi:10.3389/fpsyg.2026.1729003)
Supplement: Supplementary file 1 [file Supplementary_Figures.DOCX]

Supplementary Information


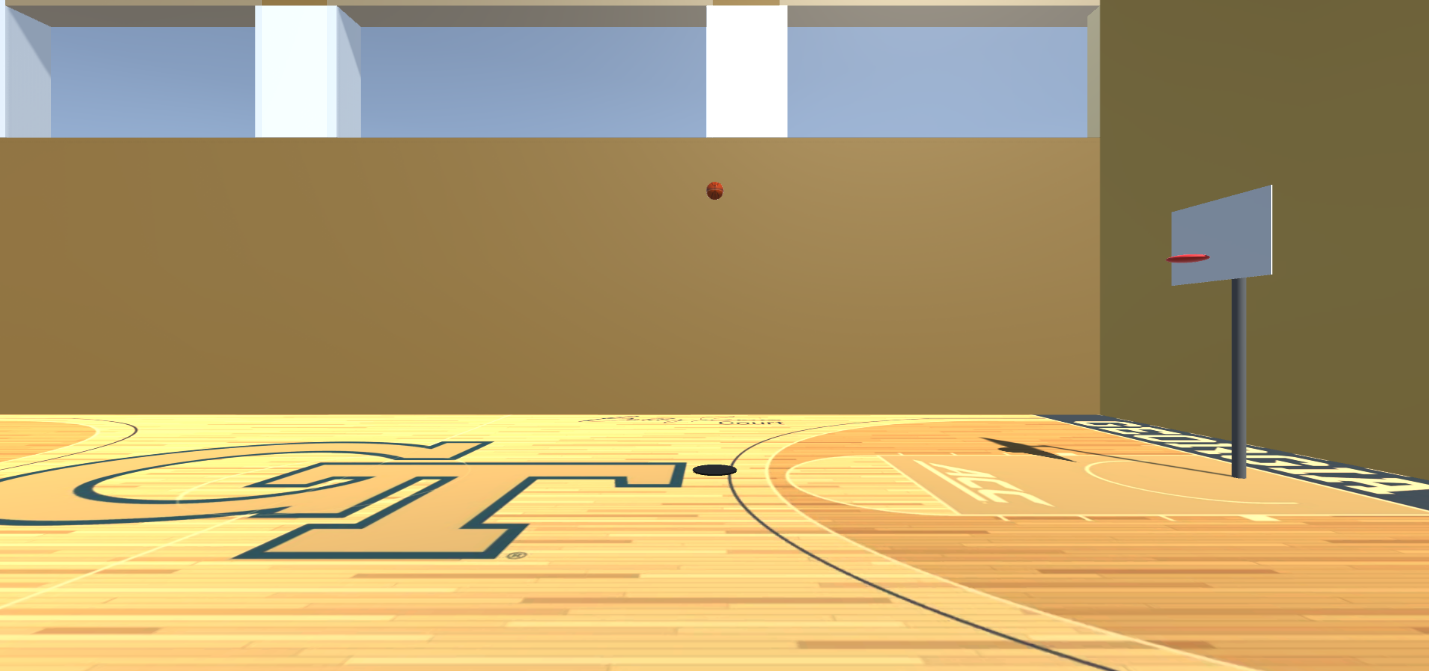


Supplementary Figure 1. Example image of virtual environment for cognitive task.


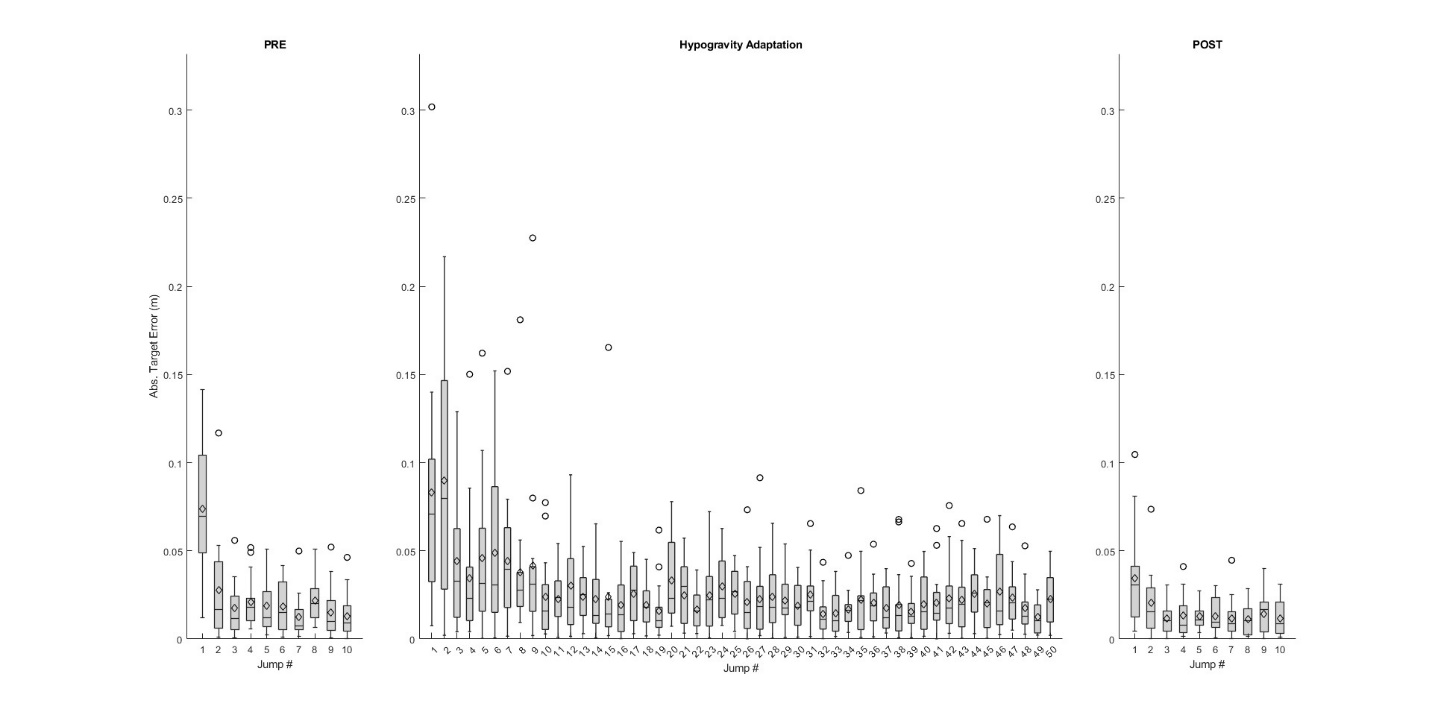


Supplementary Figure 2. Absolute target error across all jumping trials. The ten jumps from PRE (left panel), fifty jumps during hypogravity adaptation (middle panel), and ten jumps from POST (right panel) show the change in performance over the entire experiment. Boxplots represent the median (horizontal line), mean (diamond), interquartile ranges (top and bottom of each box), and range (whiskers) across all participants. Open circles represent outlier data for each trial.


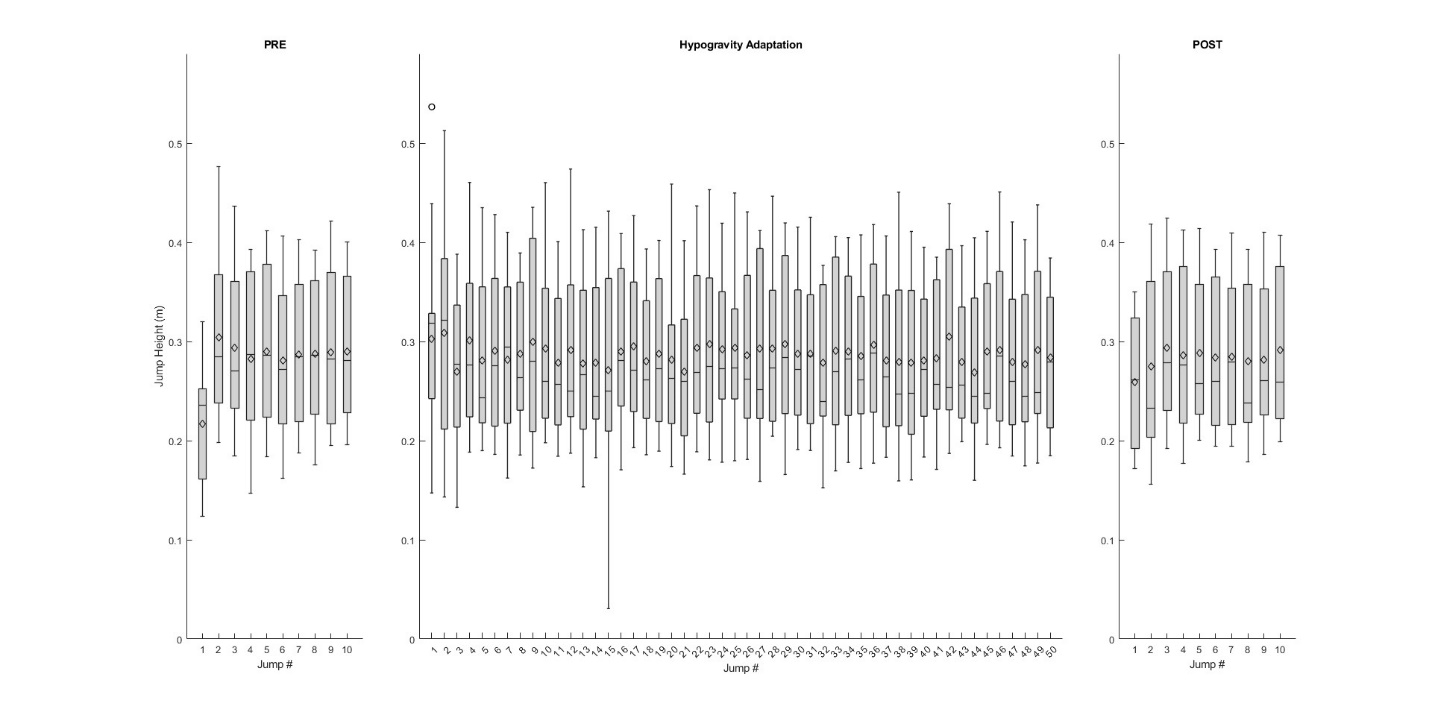


Supplementary Figure 3. Jump height across all jumping trials. The ten jumps from PRE (left panel), fifty jumps during hypogravity adaptation (middle panel), and ten jumps from POST (right panel) show the change in performance over the entire experiment. Boxplots represent the median (horizontal line), mean (diamond), interquartile ranges (top and bottom of each box), and range (whiskers) across all participants. Open circles represent outlier data for each trial.


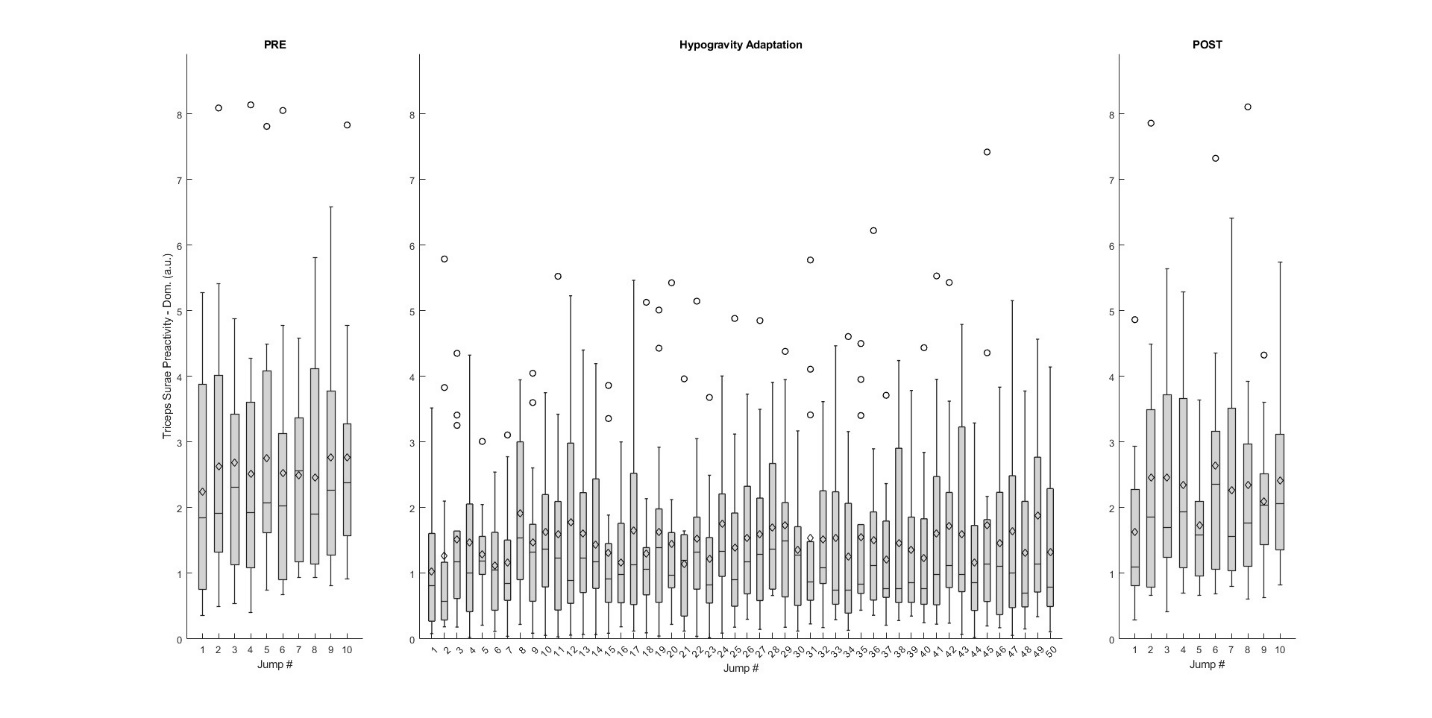


Supplementary Figure 4. Triceps Surae Preactivity of the dominant limb across all jumping trials. The ten jumps from PRE (left panel), fifty jumps during hypogravity adaptation (middle panel), and ten jumps from POST (right panel) show the change in performance over the entire experiment. Boxplots represent the median (horizontal line), mean (diamond), interquartile ranges (top and bottom of each box), and range (whiskers) across all participants. Open circles represent outlier data for each trial.


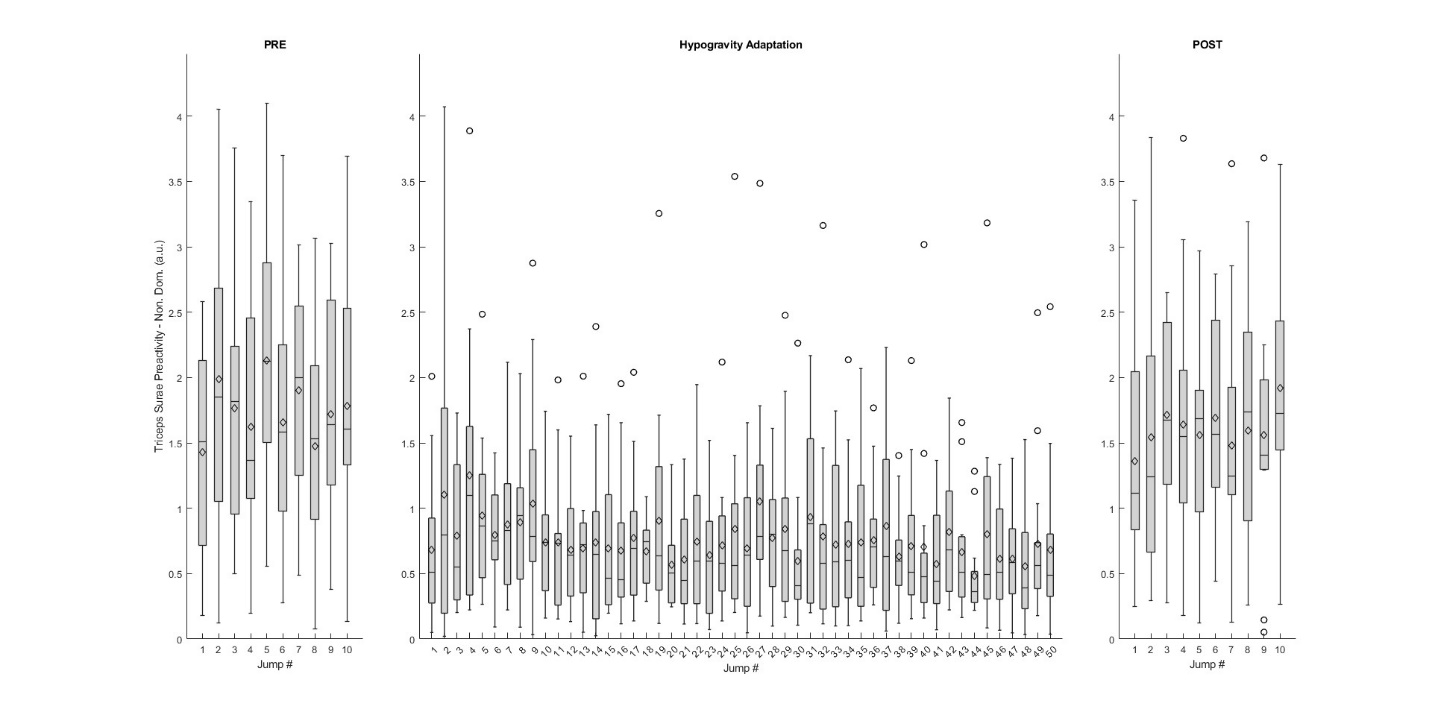


Supplementary Figure 5. Triceps Surae Preactivity of the non-dominant limb across all jumping trials. The ten jumps from PRE (left panel), fifty jumps during hypogravity adaptation (middle panel), and ten jumps from POST (right panel) show the change in performance over the entire experiment. Boxplots represent the median (horizontal line), mean (diamond), interquartile ranges (top and bottom of each box), and range (whiskers) across all participants. Open circles represent outlier data for each trial.
